# The Book World of Medicine and Science

**Published:** 1901-10-26

**Authors:** 


### The Book World of Medicine and Science.

Diseases of the Stomach and their Surgical Treatment. By A. W. Mayo-Robson, F.R.C.S., and B. G. A. Moynihan, M.S.Lond., F.R.C.S., with 65 illustrations. (London: Baillière, Tindall and Cox, 1901. Price 15s. net.)

Perhaps in no branch of surgery has such great progress been made during recent years as in that relating to the abdomen, and the fact that 75 consecutive operations on the stomach, including both malignant and simple cases, were attended by only one death shows that the surgeon who has made such a record is entitled to speak with great authority on the subject. The present work took its origin in the Hunterian Lectures delivered at the Royal College of Surgeons of England, in 1900, and is a full and lucid account of the latest experience of the authors in the surgical treatment of diseases of the stomach. The authors strike a true note when they say that the surgeon must not now leave the whole responsibility for the diagnosis to the physician, but must rather "go over the whole of the medical evidence and be prepared to supplement it by surgical methods should such be required to elucidate the case." But they think that in only a small percentage of cases is simple exploratory incision as a means of diagnosis "necessary or even justifiable," although in doubtful cases of cancer they advocate an early exploratory operation and opine that cancer should be dealt with surgically before a tumour is clinically recognisable. The chapter on this disease clearly proves indeed that pylorectomy is not an operation of very grave risk and that the probabilities of success are great. The subject of gastric ulcer is discussed at great length, little credence being given to the theory that embolism is a rue cause of the lesion, while hyperacidity is regarded more as an effect than a cause. Experiments are quoted showing that abrasions of the stomach did not heal spontaneously in animals rendered anæmic by bleeding, which explains the frequency of simple ulcers in anæmic women. In the section on diagnosis the importance of the locality of the tender spot as an indication of the site of the ulcer in the stomach seems to be somewhat exaggerated. It will probably astonish many to learn that whereas the mortality from gastric ulcer is 25 per cent. when treated medically, it is only 5 per cent. when operative measures are adopted. As these latter are the worst and most complicated cases we cannot wonder that the authors "feel it incumbent to urge most strongly that although cases of gastric ulcer should first be submitted to medical treatment, yet if such treatment fails to cure in a reasonable time, or if relapses occur on the resumption of solid food, then medical should give place to surgical treatment." The authors point out that it is unfair to the surgeon to hand him over moribund cases and unfair to the patient to dose him with medicine if he can only be cured by operation. The importance of gastric ulcer and its complications is made evident by the fact that the discussion of that disease occupies no less than one-third of the whole volume. It is incidentally mentioned that the experience in the South African war shows that bullet wounds of the stomach will in almost every instance recover without operative treatment; but "the further history of men so wounded has yet to be written, and evidence is not lacking, in our experience, which goes to show that such history is not uneventful." The description and illustrations of the various operations are clearly given, but many cases are quoted at unnecessarily great length. The volume is well arranged, but the index is very incomplete, which cannot fail to prove a serious drawback in a work which will chiefly be used for reference.

Anatomy, Descriptive and Surgical. By Henry Gray, F.R.S., F.R.C.S. The drawings by H. V. Carter, M.D., with additional drawings in later editions. Fifteenth Edition, edited by T. Pickering Pick, F.R.C.S., and by Robert Howden, M.A., M.B., C.M. (London: Longmans, Green, and Co. 1901. Price, 32s. net.)

Good old Gray! The guide of youth the counsellor of middle age; how many generations of students have not learned anatomy from its pages, and how many medical men have not made it their constant referee in all questions of anatomy for years after they commenced practice. Yet it comes up again, edition after edition, always as young as ever, always exciting our admiration by its lucid descriptions and vivid pictures, and always, as each edition comes out, giving us something new, something to make us feel that it keeps well up to date. The old plates which have been such a feature in this book remain, as do many of the descriptive pages, much as they were, but new illustrations have been added where they seemed to be required to elucidate the text, and a good deal that is fresh, with about sixty additional illustrations, will be found in the section on Embryology. The book deals with the whole subject of anatomy. First we have general anatomy or histology, then comes embryology (which is a really admirable account of the development of the fœtus), then the mass of the book deals with anatomy proper, bones, muscles, vessels, and nerves, and all the different regions, and finally there is surgical anatomy. It is a handsome well bound volume, full of large-sized illustrations, the vessels and nerves in many of them being coloured, and will fully maintain the high position which "Gray's Anatomy" has been accorded for so long by all who have wished for one complete volume dealing with all aspects of the subject. It is an invaluable students' handbook.

Arsenic. By Professor J. Alfred Wanklyn, M.R.C.S. (London: Kegan Paul, Trench, Trübner & Co. 1901. Price 2s. 6d.)

The Manchester epidemic of arsenical poisoning brought to the front the whole subject of arsenic, and while medical men found much to discuss in regard to the clinical aspects of the problem chemists found that the analysis of arsenically tainted organic compounds was not altogether so simple a matter as had been supposed. For many years past the author has interested himself in the chemical side of the problem, and this little book is the outcome of his researches He tells us a good deal about arsenic, but it would seem that his main object is to re-establish the efficacy of Marsh's test, and to point out the possibility that arsenic may exist, both in the human body and in such substances as beer, in some such organic combination (the organo-metallic condition of arsenic) as may render it very difficult to detect unless special precautions, which he describes, be taken. It is to be noted that the book is of a somewhat controversial nature, which to the general reader will probably detract from its interest.

